# Shoulder Torque Production and Muscular Balance after Long and Short Tennis Points

**DOI:** 10.3390/ijerph192315857

**Published:** 2022-11-28

**Authors:** André V. Brito, Diogo D. Carvalho, Pedro Fonseca, Ana S. Monteiro, Aléxia Fernandes, Jaime Fernández-Fernández, Ricardo J. Fernandes

**Affiliations:** 1 Centre of Research, Education, Innovation and Intervention in Sport and Porto Biomechanics Laboratory, Faculty of Sport, University of Porto, 4200-450 Porto, Portugal; 2 Faculty of Physical Activity and Sports Sciences, Universidad de León, 24004 Leon, Spain

**Keywords:** evaluation and training control, biomechanics, physiology, strength, tennis

## Abstract

Tennis is an asymmetric sport characterized by a systematic repetition of specific movements that may cause disturbances in muscular strength, power, and torque. Thus, we assessed (i) the torque, power, ratio production, and bilateral asymmetries in the shoulder’s external and internal rotations at 90 and 180°/s angular velocities, and (ii) the point duration influence of the above-mentioned variables. Twenty competitive tennis players performed external and internal shoulder rotations; an isokinetic evaluation was conducted of the dominant and non-dominant upper limbs before and after five and ten forehands. A higher torque production in the shoulder’s internal rotations at 90 and 180°/s was observed for the dominant vs. non-dominant sides (e.g., 63.1 ± 15.6 vs. 45.9 ± 9.8% and 62.5 ± 17.3 vs. 44.0 ± 12.6% of peak torque/body mass, *p* < 0.05). The peak torque decreased only after ten forehands (38.3 ± 15.8 vs. 38.2 ± 15.8 and 39.3 ± 16.1 vs. 38.1 ± 15.6 Nm, respectively, *p* < 0.05), but without impacting speed or accuracy. Unilateral systematic actions of tennis players caused contralateral asymmetries, evidencing the importance of implementing compensatory training. The forehand kinematic assessment suggests that racket and wrist amplitude, as well as speed, are important success determinants in tennis.

## 1. Introduction

Tennis is an intermittent high-intensity sport that has become faster over the years, with points (composed of four or five shots) lasting ~8 s followed by 10–25 s of recovery [1]. Depending on the tournament type (the best of three or five sets), a tennis game typically lasts 1–5 h with 20–30% of effective playing time [2,3]. The forehand, similar to the serve, accounts for >25% compared to other common tennis actions (e.g., the backhand and the volley), and consists of preparation, acceleration, impact, and finish phases [4]. Biomechanically, the forehand depends, predominantly, on the shoulder’s internal rotation and on its horizontal flexion and abduction, which is essential to efficient execution [5]. If consistent and powerful, it significantly influences the game; thus, it is considered a predictor of victory or defeat [6].

Ball speed is one of the most important tennis performance determinants since it reduces the time for the opponent to prepare and perform his/her action [6]. Nowadays, tennis players hit the ball at a much higher racket speed (leading to increased ball speed), which makes this aspect a major training goal [7]. Ball speed is associated with maximum dynamic force and power generated through a specific kinetic chain [8], specifically through the erector spinae, latissimus dorsi, triceps brachii, pectoralis major, and extensor carpi radialis muscle activation [9,10]. Another key performance factor in tennis is ball accuracy [10], which, together with the appropriate ball speed, can determine the success of a point, distinguishing more experienced players from lower-level players [6].

The shoulder rotator cuff strength and torque, as well as the range of motion, are essential to dynamically stabilizing this joint and increasing the ball speed [11,12,13]. Joint stabilization is affected by different factors (e.g., neuromuscular fatigue [14]) and it evidences a decrease in muscle strength or power capacity when it is not perfect [8], meaning that increased shoulder stability allows the sustenance of power production throughout the game [15]. Therefore, the lack of power and/or accuracy in the upper limb actions, due to peripheral fatigue (which might lead to a performance decrease [16]), is a relevant tennis research topic. In fact, some tennis-related studies have focused on performance decreases (connected with the serve, groundstroke accuracy, and velocity) and electromyographic changes associated with fatigue [17,18,19].

Inter-limb asymmetry is also a very important issue in tennis, specifically when referring to performance or functional differences between limbs [20]. These dissimilarities may be due to asymmetric motor demands throughout the game, resulting in functional asymmetric adaptions (e.g., greater muscle mass in the dominant upper limb [21,22] and unbalanced external/internal rotation ratios [23]). Furthermore, larger inter-limb asymmetry, i.e., shoulder external/internal rotation ratios outside of 66–75% or bilateral differences above 10–15%, may increase the risk of non-traumatic injuries for players [11,24]. These injuries are caused by concurrent training and competition workloads [25], altering the shoulder’s range of motion, and imbalanced muscle strength [26,27]. Even though isokinetic evaluations are mainly used in clinical contexts [13,28], they can assess the muscle torque, power, and ratio of an isolated joint [29], particularly in tennis, which has a high rate of shoulder, elbow, and wrist chronic injuries [30].

Scientific literature studies related to shoulder strength and the power assessment of the isokinetic dynamometer are available, but are limited in regard to tennis practice and match moments (i.e., on the tennis court). So, the objectives of this current study are to (i) characterize the production of torque, ratio, and bilateral asymmetries in the shoulder’s external and internal rotations; (ii) analyze the influence of point duration (five vs. ten forehand actions) in the above-referred variables, and (iii) assess the player’s forehand kinematics, ball speed, and accuracy. It was hypothesized that (i) a player’s dominant upper limb would present higher torque and power than a non-dominant limb; (ii) the torque and power production would decrease after performing five and ten forehands; (iii) the fatigue generated by the forehand actions would lead to a decrease in the shoulder internal rotation torque and power, balancing the external/internal rotation ratios; and finally (iv) ball speed and accuracy would decrease, and racket horizontal displacement velocity would increase, from five to ten forehands.

## 2. Materials and Methods

### 2.1. Participants

Fifteen male and five female competitive tennis players voluntarily participated in the current study (their general and specific characteristics are described in Table 1). All players were healthy, had no previous injuries, competed at national and international levels, and were positioned in the best national rankings of their age groups. Players were informed about the experimental procedures and possible risks involved and signed (or their respective parents signed) a consent form to participate. The host’s faculty ethics committee approved the study design, and the experimental protocol was conducted in accordance with the Declaration of Helsinki.

### 2.2. Experimental Moments

**Figure 1:**
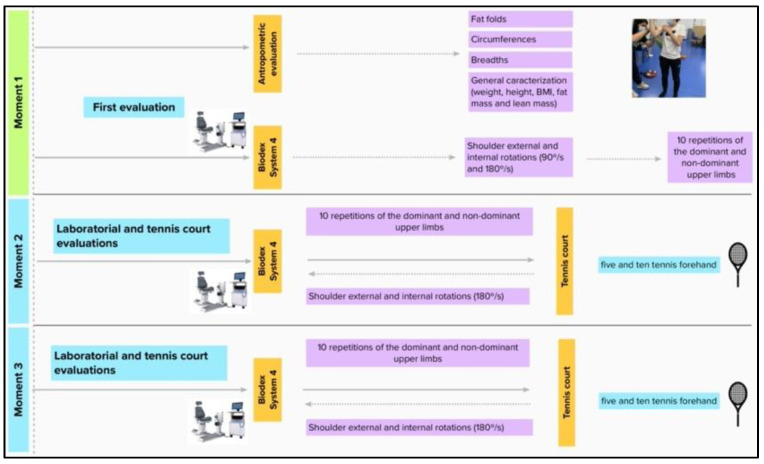
Experimental moments at the laboratory and the tennis court.

**Figure 2:**
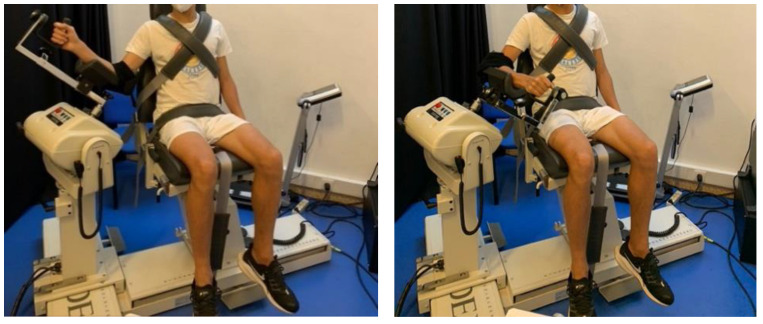
Player positioning during the shoulder internal and external rotations performed on the isokinetic dynamometer (left and right panels, respectively).

The experiments described in Figure 1 were completed without vigorous efforts in the prior 24 h. In the first moment, we conducted a general anthropometric characterization using an adipometer (Holtain Skinfold Caliper, UK), a metric tape (Sanny, Brazil), a stadiometer (Seca 222, Belgium), and a body composition (InBody 120, Korea) in accordance with the recommendations of the International Society for the Advancement of Kinanthropometry (ISAK). Then, players executed a standardized warm-up consisting of 5 min of elastics exercises and 10 min of dynamic stretching [31]. Five submaximal shoulder rotations in an isokinetic dynamometer (Biodex System 4, Biodex Medical Systems, NY, USA) were performed for familiarization, and immediately, ten shoulder external and internal rotations of the dominant and non-dominant upper limbs were performed at 90 and 180°/s (with 3 min intervals between test velocities; Figure 2) [32]. These evaluations allowed us to obtain the isokinetic variables (e.g., torque) for each experimental condition. The following equation allowed for determining the peak torque symmetry index [33]:

(1) $$\mathrm{SI}\;(\%)\;=\frac{\left(\mathrm{dominant}\;-\;\mathrm{nondominant}\right)}{0.5\;*\;(\mathrm{dominant}\;+\;\mathrm{nondominant})}*100$$

**Figure 3:**
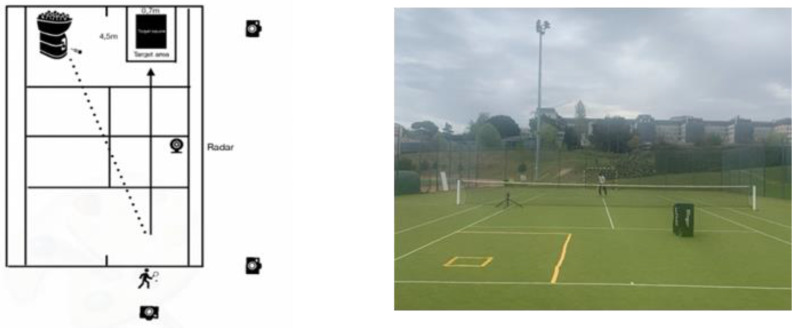
Experiments conducted on the tennis court and the respective ball machine, marking tapes, video cameras, and radar (left and right panels).

In the second and third moments (48 h apart), players performed, in a randomized order, five and ten forehands on the court, simulating a short and long point, respectively. Before and after the court test, players executed ten shoulder external and internal rotations of the dominant and non-dominant upper limbs only at 180°/s (the nearest angular velocity to the forehand made on the court). As described in Figure 3, players were positioned behind the baseline and performed the forehands as fast as possible, in square stance positions, into a 3.0 × 4.5 m area, after five minutes of court warm-ups. New tennis balls (Wilson US-Open, Chicago, IL, USA) were launched through a ball machine (Slinger bag, Windsor Mill, MD, USA) at a 20 m·s^−1^ constant speed [34] and a radar (Stalker Radar Pro II, Richardson, TX, USA) measured the forehand ball speed. The forehand kinematics (e.g., body angles) were recorded at 240 Hz by a calibrated video camera (Go-Pro Hero 7, San Mateo, CA, USA), using anatomical body markers (i.e., wrist, hip, and shoulder) [35]. A video camera with the same characteristics was used to accurately analyze; 0, 1, or 2 points were given when the ball did not enter the area, entered the large area, or entered the small area, respectively [34].

### 2.3. Statistical Analysis

Descriptive statistics are presented as the means and standard deviations (SD), and data normality and variance equality were checked through the Shapiro–Wilk test. A *t*-test of independent measures was applied to compare the general characteristics of the male and female players, and a *t*-test of repeated measures was used to compare dominant and non-dominant upper limbs regarding the dynamometric and kinematic variables. The effect size (Cohen’s d) was calculated to communicate the significance of the practical results (small effect size = 0.2; moderate effect size = 0.5 and large effect size = 0.8) and the Pearson correlation was used to analyze the connections between the studied variables. Statistical analyses were carried out using SPSS (SPSS 27.0 version, Chicago, IL, USA) and an α = 0.05 significance level was accepted.

## 3. Results

Table 2 displays the isokinetic variable values of the shoulder’s external and internal rotations at 90 and 180°/s of the first experimental moment. We observed a higher peak torque, average power, total work, and maximal repetition of total work, as well as lower external/internal rotation ratios, in the dominant upper limb’s internal rotation. Moreover, a higher peak torque and average power were present at 90°/s, while a higher average peak torque was observed at 180°/s in the dominant upper limb external rotation. Additionally, total work, maximal repetition total work, range of motion, average power at 90 and 180°/s, and average peak torque at 90°/s values were similar between dominant and non-dominant upper limb external rotations. Lastly, tennis players showed a higher peak torque and total work at 90°/s as well as a higher average power at 180°/s.

Table 3 shows the isokinetic variable values of the shoulder’s external and internal rotations before and after the five and ten forehands. Tennis players showed a higher peak torque, average power, and total work for the dominant upper limb when compared with the non-dominant upper limb. After five forehands, the results showed a decrease in the peak torque/body weight in the internal rotation and peak torque/body weight, total work, and average power for external rotation of the dominant upper limb. After ten forehands, tennis players showed a decrease in the peak torque, peak torque/body weight, total work, average power, and range of motion in the dominant upper limb and lower total work, average power, average peak torque, and range of motion. However, a similar peak torque, range of motion, and external/internal rotation ratios were registered for the dominant upper limb. In addition, the tennis players presented a lower peak torque, total work, and range of motion for the non-dominant upper limb after ten forehands.

**Figure 4:**
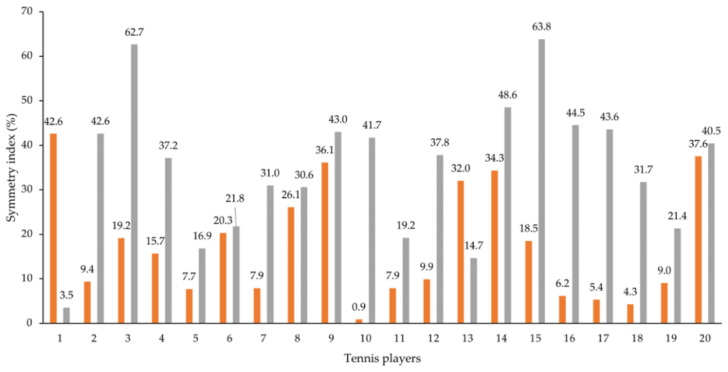
Individual peak torque symmetry indexes of the shoulder’s external and internal rotations (in orange and grey, respectively) between the dominant and non-dominant upper limbs.

**Figure 5:**
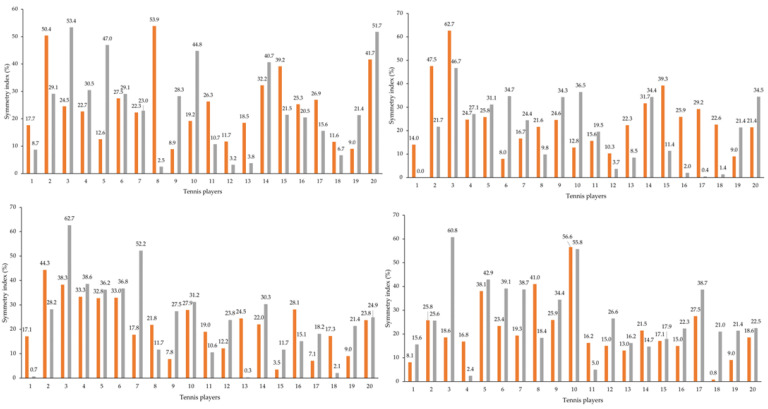
Individual peak torque symmetry indexes of the shoulder’s external and internal rotations (in orange and grey, respectively) between the dominant and non-dominant upper limbs, before and after five (higher left and right panels, respectively), and ten forehands (lower left and right panels, respectively).

Figure 4 shows the individual shoulder symmetry indexes: ten players evidenced asymmetry (>10%) between dominant and non-dominant upper limb external rotations and 19 players evidenced the same with internal rotation. Figure 5 represents those indexes before and after five and ten forehands. After 5 forehands, 17 players showed asymmetry between dominant and non-dominant upper limb external rotations and 18 players showed the same in internal rotations (higher left and right panels, respectively). After 10 forehands, 17 players presented asymmetry between the dominant and non-dominant upper limbs in the external rotation, and 18 players presented in the internal rotation (lower left and right panels, respectively). Finally, we observed that in the second and third moments, there were more players with asymmetries in the external rotation, unlike what was registered for the internal rotation, in which the values were similar. Furthermore, the first moment verified higher values with asymmetry (>10%) when compared with the two moments.

**Figure 6:**
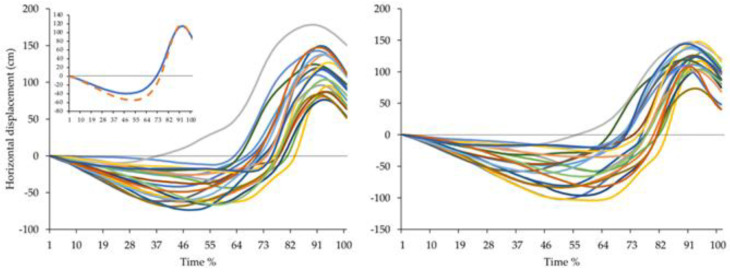
Example of the racket horizontal displacement average pattern. The five and ten forehands are represented on the left and right panels, respectively, with the average pattern of the two moments displayed in the upper left corner (orange and blue, respectively) of the left panel.

In Table 4, the five and ten forehand speeds/accuracies and kinematics are compared. After ten forehands, the average ball speed was lower, compared with five forehands; the accuracy was similar in both moments. During the forehand action, analyzed also in the graphic in Figure 6, the forearm flexion and wrist angles (before the swing and at the final phase, respectively) increased, but no differences were observed on the other kinematics angles, between five and ten forehands. Moreover, after ten forehands, increases in the horizontal racquet displacement, and the vertical racquet, shoulder, wrist, and hip displacement were observed. We observe an increase in the horizontal velocity in the racquet and wrist as well as in similar ball speeds and accuracies, as well as in horizontal displacement and velocity of the wrist, hip, and shoulder.

## 4. Discussion

In this study, we hypothesized that the (i) torque and power would present higher values in the dominant limb than in the non-dominant upper limb; (ii) torque and power production would decrease after five and ten forehands; (iii) fatigue generated by the forehand actions would lead to a shoulder internal rotation torque and power decrease, balancing the external/internal rotation ratios; and (iv) ball speed and accuracy would decrease from five to ten forehands and the racket would show higher horizontal displacement velocities. Our main findings evidenced a higher peak torque, total work, and power in the dominant upper limb; lower total work, maximal repetition total work, average power, average peak torque, and range of motion were registered after ten forehands. These main results need to be carefully interpreted because depending on the isokinetic angular velocity pre-defined, the results may be different (e.g., a higher angular velocity leading to higher power values) [36].

Since tennis is an asymmetric sport, it is important to analyze the differences between the dominant and non-dominant limbs, as well as player anthropometrics to characterize this specific population [37]. Our male players presented similar height, body mass, BMI, triceps/biceps skinfold, and relaxed/tensed arm perimeter values to other studies (176.8 ± 6.4 cm, 69.9 ± 6.8 kg, 22.3 ± 1.4 kg/m^2^, 9.5 ± 2.7/4.3 ± 1.2 mm and 28.7 ± 1.7/30.7 ± 1.8 cm) [37]. However, female players showed lower values when compared to the reference literature (165.4 ± 6.3 cm, 59.9 ± 6.2 kg, 21.9 ± 1.7 kg/m^2^, 16.3 ± 4.0/7.4 ± 2.6 mm and 27.0 ± 1.8/27.8 ± 1.7 cm) [38]. Furthermore, height and body mass values presented in the literature for males and females (174.2 ± 7.6, 64.3 ± 7.9, and 164.9 ± 3.7 cm, 50.1 ± 6.7 kg, respectively) [39] were also lower when compared with the current study. We verified in other studies that male players trained more hours per week than in this study (29.0 ± 6.7 h) [40], which can be attributed to age, sex, and level differences.

Long-term accumulated loads in different sports can lead to body asymmetries and upper-dominant limb dominance [41,42]. The results of torque, power, and total work showed that the dominant upper limb internal rotations (forehand action) were more proficient compared to those of the non-dominant. In comparison with other studies, it was evidenced that our results (between the dominant and non-dominant upper limb internal rotations) were similar to those of other competitive tennis players (peak torque: 42.6 ± 9.5 vs. 35.8 ± 8.4 N.m, mean power: 92.2 ± 26.5 vs. 76.4 ± 21.4 W, single repetition work: 30.0 ± 7.0 vs. 19.2 ± 8.0 J) [35,43]. Moreover, our study showed higher peak torque and power for the dominant external rotation and a lower maximal repetition total work in the non-dominant limb when compared with those same studies (peak torque: 33.1 ± 6.1 vs. 29.7 ± 6.8 N.m, mean power: 70.6 ± 18.6 vs. 60.1 ± 18.0 W, maximal repetition total work: 22.1 ± 6.1 vs. 23.6 ± 8.0 J).

Throughout a tennis game, peripheral fatigue caused by unilateral and repeated tennis movements can cause disturbances in the player’s performance [16,26,44]. During competitive situations, the number of forehands can vary between 4 and 10, according to the duration of the point, and after the serve, is the most relevant action, in which the shoulder’s internal rotation is also important. This information can be very useful to coaches in order to provide information about the overload in the shoulder function. Results obtained in the current study showed that, after a long point (e.g., ten forehands), there was a reduction in the range of motion, total work, average peak torque, and power production in external/internal rotations, with more magnitude in the dominant internal rotation (forehand action). When compared with other studies, it was possible to verify that the isometric strength in the external rotation (−5.8%) and dominant side range of motion in males (−8.3%) and females (−6%) decreased after a game [45]. 

The internal rotator muscles are considered the agonist muscles of motion [21], being solicited in the acceleration phase of tennis forehands [46]. The external/internal rotation ratios in the first experimental moment showed a weakness of the antagonist muscles over the agonist ones for both limbs, which is in line with the results obtained in a previous study, (external/internal rotation ratio: 79.0 ± 14.0 vs. 83.0 ± 10.0% and 68.0 ± 7.0 vs. 96.0 ± 18.0%) [43]. After five and ten forehands, the external/internal rotation ratios increased, compared with the first isokinetic moment, which is in accordance with the asymmetry acceptable range (66–75%). The increase of the external/internal rotation ratios is justified by the fatigue and consequent loss of strength in the dominant upper limb internal rotation after the forehands, despite the unchanged strength in the external rotation. These ratios also changed in players in combination with a series of ten serves (dominant and non-dominant upper limb external/internal rotation ratios: 79.0 ± 0.12 vs. 83.0 ± 0.13%) [12]. 

Several biomechanical factors (e.g., racket acceleration) were found to justify the success of the tennis forehand [4,6]. According to previous research, the shoulder’s external and internal rotations and racket horizontal velocity contribute between 40 and 54% [47] and 94% [5] to the whole forehand, respectively. In the present study, racket and wrist displacements and horizontal velocity were shown to be greater than those of the shoulder and hip. In ten forehands, the horizontal and vertical displacements were higher, compared to the effects registered after five forehands. These displacement results are in line with previous studies, with values similarly high in the upper limb horizontal displacement when moving the racket from the beginning to the end of the movement [4]. Moreover, the horizontal velocity values also showed similarities in the racket and hip horizontal velocity (15.4 ± 1.4 and 1.5 ± 0.3 m·s^−1^, respectively), but lower values in the wrist and shoulder horizontal velocity (10.0 ± 1.1 and 2.4 ± 0.3 m·s^−1^, respectively) [6,34].

Complementarily, the wrist angle had the highest value recorded in the forehand preparation and end phase, showing similarities with other studies (137.0 ± 0.2 vs. 143.5 ± 0.5°). The angles generated by the dominant upper limb abduction and the racket with the ball were higher than in other studies (32. 0 ± 12.0, 80.6 ± 3.6°), in contrast to the forearm flexion angles in the forehand hitting and finishing phases, which were lower compared to other studies (137.5 ± 0.1 and 131.7 ± 0.5°) [6,47,48]. Since kinematic analysis is very important in tennis, forehand outcomes (e.g., ball speed and accuracy) have become important research factors [34]. The results showed that the ball speed was lower after ten forehands and the accuracy was similar in both moments when compared with the forehands of other tennis players from several other studies [28,34].

In this study, the peak torque symmetry index between the dominant and non-dominant upper limbs showed that the shoulder’s external and internal rotations presented abrupt asymmetry between limbs (e.g., before the five forehands, the external and internal rotations showed values of 30.5 and 22.7%, respectively). It was also evident that after the ten forehands, some players presented lower symmetry indexes due to the unused non-dominant upper limb and the lack of dominant limb strength after use, which decreased the index. In comparison with other studies, it was verified that the dominant upper limb’s external and internal rotations also presented high symmetry index values (external/internal rotation symmetry index: 33.0/34.4%) [49].

The current study has several limitations, including (i) the absence of a comparison between genders and age groups; (ii) specific tennis point durations were used when they varied along the game; (iii) only the forehand was evaluated, which is one of several actions that tennis players use; and (iv) the dynamometer speed only reached 300°/s, which is dissimilar from the speed performed by the racket. Future research is needed to address these issues, improve the characteristics of tennis, and differentiate between male and female players

## 5. Conclusions

Our objective was to understand the influences of short and long tennis points on isokinetic variables. Our data allow us to conclude that five and ten forehands lead to an increase in the external/internal rotation ratios (but without improvement in joint stability) and to a decrease in the torque, power, and total work values. Moreover, the dominant vs. non-dominant upper limbs presented higher torque and power in external and internal rotations (evidencing the bilateral asymmetrical characteristics of tennis players). It is extremely important to use the maximum amplitude of the limb to increase the forehand power, and employ the maximum horizontal velocity of the racket and wrist. Finally, conducting an isokinetic evaluation in specific tennis match conditions can help coaches improve the training process and achieve their predefined goals.

## Figures and Tables

**Table 1:** Mean (SD) values of age, training background, and main anthropometric characteristics.

Variables	Male	Female	Total
Age (years)	20.1 (5.4) *	14.4 (0.5)	18.7 (5.3)
Training frequency (sessions/week)	5.1 (1.5)	5.0 (0.0)	5.1 (1.3)
Training volume (h/week)	10.7 (2.7)	10.0 (0.0)	10.5 (2.4)
Height (cm)	177.2 (8.2)	161.7 (4.4)	173.7 (10.0)
Body mass (kg)	67.0 (10.8) *	48.9 (4.3)	62.5 (11.7)
Body mass index (kg/m^2^)	21.3 (2.0) *	18.7 (1.2)	20.6 (2.2)
Biacromial breadth (cm)	39.2 (2.9) *	35.4 (1.6)	38.3 (3.1)
Bicristal breadth (cm)	27.2 (1.9) *	24.3 (1.3)	26.5 (2.2)
Humeral breadth (cm)	6.6 (0.3) *	6.0 (0.5)	6.5 (0.4)
Wrist breadth (cm)	5.4 (0.2) *	4.8 (0.3)	5.2 (0.3)
Palmar transverse breadth (cm)	20.6 (3.5)	19.8 (1.5)	20.4 (3.1)
Palmar longitudinal breadth (cm)	19.4 (1.3) *	17.9 (0.6)	19.1 (1.3)
Stretched arm circumference (cm)	30.3 (3.2) *	24.8 (0.7)	28.9 (3.7)
Relaxed arm circumference (cm)	28.6 (2.8) *	23.8 (0.7)	27.4 (3.2)
Forearm circumference (cm)	25.8 (2.2) *	22.5 (1.5)	25.0 (2.5)
Upper arm length (cm)	57.2 (2.4) *	50.9 (1.9)	55.7 (6.2)
Upper arm length (cm)	26.6 (3.4) *	23.5 (0.7)	25.9 (3.3)
Arm length (cm)	30.2 (1.9)	28.8 (1.4)	29.9 (1.9)
Bicipital folds (mm)	5.5 (1.5) *	6.8 (1.6)	5.5 (1.7)
Tricipital folds (mm)	10.2 (3.5) *	13.4 (1.7)	11.0 (3.4)

* Differences between male and female players.

**Table 2:** Mean (SD) values of torque, power, total work, range of motion, and internal/external rotations (IR and ER) ratios at 90 and 180°/s angular velocities in the dominant and non-dominant upper limbs.

Variables		Dominant Upper Limb	Non-Dominant Upper Limb	*t* Test		Cohen’s d
				*t*	*ρ*	
90°/s angular velocity						
Peak torque (N.m)	ER	21.9 (6.6)	20.2 (6.3)	2.56	0.01 *	0.57
	IR	40.0 (14.9)	29.1 (10.1)	6.45	0.00 *	1.44
Peak torque/body weight (%)	ER	34.9 (6.1)	32.1 (5.9)	2.83	0.01 *	0.63
	IR	63.1 (15.6)	45.9 (9.8)	7.28	0.00 *	1.63
Total work (J)	ER	263.2 (84.7)	243.3 (88.0)	1.38	0.18	0.30
	IR	541.6 (224.9)	390.2 (143.2)	4.74	0.00 *	1.06
Maximal repetition total work (J)	ER	30.3 (9.6)	27.3 (9.4)	1.88	0.07	0.42
	IR	62.3 (23.0)	44.6 (15.3)	5.30	0.00 *	1.18
Average power (W)	ER	18.3 (6.5)	17.0 (7.1)	1.40	0.00 *	0.31
	IR	37.4 (7.1)	27.0 (11.6)	5.26	0.00 *	1.17
Average peak torque (N.m)	ER	19.1 (5.9)	18.0 (6.3)	1.49	0.15	0.33
	IR	35.3 (14.5)	25.8 (9.7)	5.78	0.00 *	1.29
Range of motion (°)		121.2 (11.9)	120.7 (12.8)	0.24	0.80	0.05
External/internal rotation ratios (%)		57.7 (13.2)	70.9 (10.6)	−5.26	0.00 *	1.17
180º/s angular velocity						
Peak torque (N.m)	ER	21.4 (7.9)	19.1 (6.8)	2.15	0.04 *	0.48
	IR	39.9 (16.4)	28.1 (11.5)	6.71	0.00 *	1.50
Peak torque/body weight (%)	ER	33.7 (7.8)	29.9 (6.0)	2.45	0.02 *	0.55
	IR	62.5 (17.3)	44.0 (12.6)	8.15	0.00 *	1.82
Total work (J)	ER	219.8 (84.3)	193.6 (89.0)	2.07	0.05	0.46
	IR	534.4 (244.5)	363.8 (161.3)	5.36	0.00 *	1.20
Maximal repetition total work (J)	ER	25.1 (8.9)	23.0 (9.7)	1.46	0.15	0.32
	IR	60.3 (25.4)	41.8 (17.7)	5.60	0.00 *	1.25
Average power (W)	ER	25.4 (10.9)	22.4 (12.1)	1.90	0.07	0.42
	IR	63.3 (33.2)	42.1 (22.2)	5.22	0.00 *	1.16
Average peak torque (N.m)	ER	19.0 (7.0)	16.6 (6.4)	2.40	0.02 *	0.53
	IR	36.2 (15.7)	25.1 (10.4)	6.24	0.00 *	1.39
Range of motion (°)		122.5 (10.5)	124.1 (12.8)	−0.99	0.33	0.22
External/internal rotation ratios (%)		56.7 (16.2)	70.6 (13.4)	−5.90	0.00 *	1.32

* Differences between dominant and non-dominant upper limbs.

**Table 3:** Mean (SD) values of torque, power, total work, range of motion, and internal (IR)/external rotation (ER) ratios between dominant and non-dominant upper limbs before and after five and ten forehands.

Variables		Dominant Upper Limb				Non-Dominant Upper Limb			
		Before Five Forehands	After Five Forehands	Before Ten Forehands	After Ten Forehands	Before Five Forehands	After Five Forehands	Before Ten Forehands	After Ten Forehands
Peak torque (N.m)	ER	26.4 (9.3)	26.3 (8.3)	27.4 (8.5)	26.6 (9.1)	21.1 (6.9) ^4^	20.9 (7.1) ^4^	21.3 (6.3) ^4^	22.1 (5.7) ^4^
	IR	38.3 (15.8)	38.2 (15.8)	39.3 (16.1)	38.1 (15.6) ^3^	30.5 (12.0) ^4^	31.0 (12.1) ^4^	30.0 (12.5) ^4^	30.4 (12.3) ^1,4^
Peak torque/body weight (%)	ER	42.5 (10.7)	42.0 (8.5) ^2^	43.9 (9.3)	42.7 (10.6)	34.1 (7.3) ^4^	33.5 (7.9) ^4^	34.1 (6.9) ^4^	35.7 (5.1) ^4^
	IR	60.3 (16.7)	59.5 (16.1) ^2^	61.5 (16.9)	60.4 (16.8) ^3^	48.5 (13.6) ^4^	49.0 (13.3) ^4^	47.5 (14.6) ^4^	48.4 (13.2) ^4^
Total work (J)	ER	273.4 (108.0)	265.6 (94.6) ^2^	263.7 (91.4)	238.8 (84.0) ^1,3^	223.5 (83.4) ^4^	225.4 (85.1) ^4^	226.9 (85.9) ^4^	214.8 (72.9) ^1^
	IR	580.9 (285.4)	584.1 (268.5)	579.6 (259.8)	529.9 (265.9) ^1,3^	403.5 (197.9) ^4^	416.4 (192.5) ^2,4^	394.1 (184.4) ^4^	385.2 (174.6) ^4^
Maximal repetition total work (J)	ER	30.7 (12.1)	29.6 (10.6) ^2^	29.3 (10.1)	26.5 (9.2) ^1,3^	24.7 (8.9) ^4^	25.2 (9.2) ^4^	25.3 (9.0) ^4^	24.2 (8.4)
	IR	65.4 (29.4)	65.1 (28.4)	66.0 (28.2)	60.5 (29.3) ^3^	45.8 (21.1) ^4^	47.3 (20.5) ^4^	44.9 (19.7) ^4^	44.3 (19.6)^4^
Average power (W)	ER	31.8 (14.7)	48.1 (30.2) ^2^	30.3 (11.9)	28.1 (11.8) ^1,3^	26.0 (11.4) ^4^	25.7 (11.5) ^4^	26.7 (11.9) ^4^	25.8 (10.9)
	IR	69.8 (37.5)	67.8 (34.4)	68.6 (34.2)	63.9 (35.4) ^3^	47.0 (25.6) ^4^	47.5 (24.5) ^4^	46.4 (24.1) ^4^	46.4 (23.3) ^4^
Average peak torque (N.m)	ER	22.6 (8.3)	22.9 (7.7)	23.3 (7.4)	22.8 (7.7)	17.7 (5.4) ^4^	18.1 (6.2) ^4^	18.3 (5.6) ^4^	18.8 (5.1) ^4^
	IR	34.9 (15.5)	34.4 (14.8)	35.1 (14.5)	33.6 (14.2) ^1^	26.9 (11.2) ^4^	26.9 (11.2) ^4^	26.4 (11.4) ^4^	26.3 (10.9) ^4^
Range of motion (°)		124.0 (9.2)	125.9 (10.5)	124.3 (12.1)	121.6 (9.8) ^1,3^	118.7 (9.8) ^4^	121.9 (10.2) ^4^	118.8 (10.7) ^4^	116.9 (11.2) ^1,4,3^
External/internal rotation ratios (%)		71.3 (14.5)	73.2 (15.2)	71.9 (11.6)	72.8 (14.6)	73.4 (16.7)	70.6 (16.3)	74.2 (15.0)	76.1 (14.0)

^1, 2, 3,^ and ^4^ represent the differences in values registered between before and after five and ten forehands, only before and after five forehands, only before and after ten forehands, and only between the dominant and non-dominant upper limbs (*p* < 0.05), respectively.

**Table 4:** Mean (SD) values of ball speed and accuracy, and kinematic variables after five and ten forehands.

Variables	Five Forehands	Ten Forehands	*t*-Test		Cohen’s d
			*t*	*ρ*	
Ball speed (km/h)	122.2 (17.5)	120.4 (15.3)	0.95	0.35	0.21
Ball accuracy (0–2 scale) ^1^	1.0 (0.2)	1.0 (0.1)	0.29	0.76	0.06
Handgrip angle in the preparation phase (°)	134.4 (16.6)	132.1 (13.8)	0.76	0.45	0.17
Limb abduction angle in the preparation phase (°)	39.9 (10.7)	38.6 (11.9)	0.84	0.41	0.18
Ball to racket angle in the phase before the swing (°)	94.0 (5.0)	91.9 (5.3)	−0.78	0.44	−0.17
Forearm flexion angle in the phase before the swing (°)	112.1 (16.5)	114.4 (18.1)	3.23	0.00 *	0.72
Angle between the ball/racket in the phase before swing (°)	97.8 (5.6)	97.7 (4.9)	0.16	0.86	0.03
Forearm flexion angle in the final phase (°)	113.0 (11.9)	113.6 (13.1)	−0.32	0.75	0.07
Angle of the wrist in the final phase (°)	139.6 (7.6)	150.1 (9.5)	−3.35	0.00 *	0.75
Horizontal racquet displacement (cm)	160.9 (26.4)	181.1 (24.2)	−2.76	0.01 *	0.61
Vertical racket displacement (cm)	86.4 (24.8)	95.6 (16.0)	−2.51	0.02 *	0.56
Horizontal shoulder displacement (cm)	44.1 (15.2)	45.6 (16.1)	−0.50	0.62	0.11
Vertical shoulder displacement (cm)	12.9 (3.8)	17.2 (4.4)	−6.17	0.00 *	1.38
Horizontal wrist displacement (cm)	97.7 (23.5)	100.6 (15.9)	−0.49	0.62	0.11
Vertical wrist displacement (cm)	41.6 (11.7)	47.8 (9.3)	−3.44	0.00 *	0.77
Horizontal hip displacement (cm)	42.5 (18.2)	44.8 (15.7)	−0.66	0.51	0.14
Vertical hip displacement (cm)	9.6 (3.4)	11.9 (3.5)	−3.90	0.00 *	0.87
Horizontal racket speed (m·s^−1^)	16.4 (3.7)	19.5 (3.2)	−3.03	0.00 *	0.67
Horizontal shoulder speed (m·s^−1^)	1.9 (0.8)	2.1 (0.7)	−0.82	0.42	0.18
Horizontal wrist speed (m·s^−1^)	6.1 (1.5)	6.9 (1.7)	−2.37	0.02 *	0.53
Horizontal hip speed (m·s^−1^)	1.6 (0.5)	1.6 (0.5)	−0.42	0.67	0.09

* and ^1^ indicate differences between five and ten forehands and the accuracy assessment, respectively (0—the ball does not enter the area, 1—the ball enters the large area, and 2—the ball enters the small area).

## Data Availability

Raw data can be requested by sending an email to abritovilela@hotmail.com or up201902341@up.pt.
